# AMPK activation stabilizes HIF-2α by disrupting VHL binding and promotes erythroid differentiation under anemic stress via the mTOR pathway

**DOI:** 10.3389/fphys.2026.1932780

**Published:** 2026-10-16

**Authors:** Yun Cen, Pingjing Cai, Jinjin Jiang, Lihua Huang, Yiwei Hua

**Affiliations:** 1 The College of Food and Health of Guangzhou City Polytechnic, Guangzhou, China; 2 The Department of Intensive Care Unit, The First Affiliated Hospital of Jinan University, Guangzhou, China

**Keywords:** AMPK, anemia, erythroid differentiation, HIF-2α, mTOR, VHL

## Abstract

**Objective:**

Anemia affects over two billion people globally, and ESA resistance poses major clinical hurdles. HIF-2α governs erythropoiesis, yet how AMPK/mTOR energy sensors regulate HIF-2α in erythroid cells remains unclear. This study explored whether AMPK activation stabilizes HIF-2α via impaired VHL binding to boost erythroid differentiation under anemic stress through mTOR signaling.

**Methods:**

UT-7/EPO cells were cultured under 1% O_2_ with low EPO to mimic anemic stress, treated with AMPK/mTOR pharmacological modulators. CHX chase and Co-IP detected HIF-2α stability and VHL interaction; Ser672 phosphorylation, erythroid and iron-related genes, hepcidin and intracellular iron were measured.

**Results:**

Anemic stress suppressed cell viability, stabilized HIF-2α, activated AMPK and inhibited mTOR, upregulated erythroid markers and triggered apoptosis. AICAR extended HIF-2α half-life, disrupted HIF-2α-VHL binding and elevated Ser672 phosphorylation, while mTOR activator MHY1485 reversed these effects. AMPK activation elevated EPOR, DMT1, FPN1, lowered hepcidin and ferritin, and restored intracellular iron; these effects vanished upon HIF-2α knockdown or mTOR overactivation.

**Conclusions:**

AMPK stabilizes HIF-2α by blocking VHL binding to facilitate erythroid maturation and iron homeostasis under anemic stress via mTOR. The AMPK/mTOR/HIF-2α cascade controls stress erythropoiesis, and targeting this axis may treat ESA-insensitive anemia.

## 1 Introduction

Anemia, defined by a reduction in circulating hemoglobin levels and consequent impairment of oxygen-carrying capacity, currently affects approximately one-third of the global population and constitutes a major comorbid burden in patients with chronic kidney disease (CKD), heart failure, and various inflammatory conditions (Babitt and Lin, 2012; GBD 2021 Anaemia Collaborators, 2023). Although erythropoiesis-stimulating agents (ESAs) have substantially transformed the clinical management of anemia, approximately 10% of treated patients display suboptimal responsiveness to these agents, leading to persistent anemia even in the setting of high-dose erythropoietin (EPO) administration (Macdougall and Cooper, 2002; Okonko et al., 2013). The molecular basis of erythropoietin (EPO) resistance remains elusive, underscoring an urgent need to discover novel therapeutic targets capable of both promoting erythroid differentiation and optimizing iron utilization.

The hypoxia-inducible factor (HIF) pathway functions as the master transcriptional regulator of cellular adaptation to reduced oxygen availability and is centrally involved in the control of erythropoiesis (Semenza, 2012; Haase, 2013). In normoxic conditions, HIF-α isoforms—namely HIF-1α and HIF-2α—are hydroxylated by prolyl hydroxylase domain (PHD) enzymes, which promotes their recognition by the von Hippel–Lindau (VHL) E3 ubiquitin ligase and targets them for proteasomal degradation (Maxwell et al., 1999; Kaelin et al., 2008). Conversely, hypoxia suppresses PHD enzymatic activity, thereby stabilizing HIF-α proteins and enabling their nuclear translocation, where they heterodimerize with HIF-β and drive the transcriptional activation of downstream target genes, including EPO, EPOR, and key iron-metabolism regulators such as SLC11A2 (which encodes divalent metal transporter-1, DMT1) and SLC40A1 (encoding ferroportin, FPN1) (Mastrogiannaki et al., 2009; Kapitsinou et al., 2010). Notably, HIF-2α has emerged as the predominant isoform regulating erythropoiesis, as selective HIF-2α activation promotes erythroid progenitor expansion and EPO production, whereas HIF-1α primarily governs metabolic adaptation (Kondo et al., 2003; Gruber et al., 2007; Lima et al., 2025).

The AMP-activated protein kinase (AMPK) and mechanistic target of rapamycin (mTOR) pathways operate as principal energy-sensing nodes that integrate cellular metabolic status with growth factor signaling (Hardie et al., 2012; Saxton and Sabatini, 2017). Under conditions of energy stress—characterized by a low ATP: AMP ratio—AMPK becomes activated, promoting catabolic metabolism while concomitantly suppressing anabolic processes, notably through inhibition of mTOR complex 1 (mTORC1) (Inoki et al., 2012). Although the AMPK/mTOR axis has been linked to diverse hypoxia-adaptive responses, its functional involvement in erythropoiesis remains inadequately defined. Accumulating evidence, however, indicates that AMPK activation may influence HIF stability via both canonical PHD/VHL-dependent mechanisms and alternative, non-canonical pathways. For instance, AMPK has been shown to directly phosphorylate HIF-1α at Ser-777/Ser-778, enhancing its transcriptional activity independently of oxygen tension (Moulin et al., 2025). However, whether AMPK similarly regulates HIF-2α in erythroid cells, and how this interacts with mTOR signaling, has not been systematically investigated.

The functional crosstalk among AMPK, mTOR, and HIF-2α assumes particular significance under anemic stress conditions, wherein erythroid progenitor cells are concurrently confronted with hypoxia, restricted erythropoietin (EPO) availability, and disrupted iron homeostasis. Inflammatory cytokines, which are frequently elevated in anemic patients, can activate AMPK while suppressing mTOR signaling, potentially creating a metabolic environment that influences HIF-2α stability and downstream target gene expression (Stenvinkel and Bárány, 2002; Khalil et al., 2016). Moreover, clinical studies have demonstrated that EPO resistance correlates with elevated inflammatory markers and impaired JAK-STAT signaling downstream of the EPO receptor (EPOR) (Cooper et al., 2003; Cen et al., 2022), suggesting that defects at the level of erythroid progenitors—rather than simply inadequate EPO production—contribute to anemia pathophysiology.

Despite these observations, several fundamental questions remain unanswered. First, does AMPK activation directly modulate HIF-2α protein stability in erythroid precursor cells? Second, is this effect mediated through canonical VHL-dependent degradation or through alternative mechanisms? Third, how does mTOR activity, as a downstream effector of AMPK, influence HIF-2α function? Finally, does pharmacological modulation of the AMPK/mTOR axis impact erythroid differentiation and iron-regulatory gene expression under anemic stress conditions? To address these questions, we established a cell-based model of anemic stress using UT-7/EPO cells—a human erythroid cell line that is strictly dependent on EPO for survival and proliferation—cultured under hypoxic conditions (1% O_2_) with limited EPO (0.2 U/mL). This model successfully recapitulated key features of anemia-associated bone marrow stress, including time-dependent suppression of cell proliferation (52.4% inhibition at 24 h), HIF-2α stabilization (8.0-fold increase), AMPK activation (3.4-fold increase in p-AMPK/AMPK ratio), reciprocal mTOR suppression (0.56-fold decrease in p-mTOR/mTOR ratio), induction of erythroid differentiation markers (EPOR: 2.7-fold; γ-globin: 3.2-fold), and activation of apoptotic pathways (Cleaved Caspase-3: 5.6-fold; Bax/Bcl-2 ratio: 5.6-fold).

Using this experimental system, we examined the impact of AMPK and mTOR pathway modulation on HIF-2α protein stability, its interaction with VHL, as well as the transcriptional expression of EPOR and key iron-metabolism-related genes. Our findings demonstrate that AMPK activation significantly prolongs HIF-2α half-life from 4.2 h to 8.5 h by disrupting VHL binding, an effect that is associated with increased phosphorylation of HIF-2α at Ser672. Conversely, mTOR activation promotes HIF-2α degradation (half-life reduced to 1.5 h) and suppresses downstream target gene expression, indicating that the AMPK/mTOR pathway serves as a critical rheostat controlling HIF-2α stability. Functionally, AMPK activation enhances EPOR, DMT1, and FPN1 expression, reduces hepcidin secretion (from 25.3 ng/mL to 12.4 ng/mL) and ferritin levels (from 0.85 to 0.52), and partially restores intracellular iron levels (from 68% to 85% of control), thereby promoting erythroid differentiation and improving iron availability under anemic stress conditions. Collectively, these findings establish AMPK as a critical proximal modulator of HIF-2α protein stability within the erythroid compartment, and further point to the AMPK/mTOR/HIF-2α signaling cascade as a promising pharmacological target for the alleviation of anemia, particularly in patients who demonstrate suboptimal responses to ESA therapy.

## 2 Materials and methods

### 2.1 Cell culture and anemic stress model

The UT-7/EPO human erythroid cell line (DSMZ, ACC 363) was cultured in Iscove’s modified Dulbecco’s medium (IMDM; Gibco, 12440053) supplemented with 10% fetal bovine serum (FBS; Gibco, 10270106), 1% penicillin–streptomycin (Gibco, 15140122), and 2 U/mL recombinant human erythropoietin (EPO; R&D Systems, 287-TC), and maintained at 37 °C in a humidified 5% CO_2_ incubator. To recapitulate anemic stress, cells were transferred to medium containing a reduced EPO concentration (0.2 U/mL) and subjected to hypoxic exposure (1% O_2_, 5% CO_2_, balanced N_2_) for 24 h within a hypoxia workstation (Invivo2 400, Ruskinn). As an alternative means of inducing chemical hypoxia, parallel cultures were treated with 100 μM cobalt chloride (CoCl_2_; Sigma-Aldrich, C8661) for the same duration.

### 2.2 Pharmacological treatments

Cells were treated with the pharmacological modulators for 30 min before being subjected to hypoxic conditions: AICAR (AMPK activator, 1 mM, Sigma-Aldrich, A9978), Compound C (AMPK inhibitor, 10 μM, Sigma-Aldrich, P5499), Rapamycin (mTOR inhibitor, 100 nM, Sigma-Aldrich, R0395), and MHY1485 (mTOR activator, 5 μM, MedChemExpress, HY-103186). All treatments were maintained throughout the 24 h hypoxia incubation period. Control cells were cultured under normoxic (21% O_2_) conditions with normal EPO (2 U/mL) for the same duration.

### 2.3 Small interfering RNA transfection

To achieve HIF-2α knockdown, UT-7/EPO cells were transfected with 50 nM siRNA specifically targeting human HIF-2α (EPAS1; Santa Cruz Biotechnology, sc-35314) or a negative control siRNA (sc-37007), using Lipofectamine 3000 (Invitrogen, L3000015) in accordance with the manufacturer’s instructions. Transfection efficiency was evaluated by Western blotting at 48 h post-transfection. Knockdown was restricted to designated experimental groups (Hypoxia + AICAR + siHIF-2α) to confirm the specificity of HIF-2α-dependent responses.

### 2.4 Cell viability assay

Cell viability was evaluated using the Cell Counting Kit-8 (CCK-8; Dojindo, CK04). Cells were plated in 96-well plates at a density of 1 × 10^4^ cells per well, with three replicate wells set up for each treatment condition. Following 24 h of treatment, 10 μL of CCK-8 reagent was added to each well, and the plates were incubated at 37 °C for an additional 2 h. The absorbance at 450 nm was recorded using a microplate reader (BioTek, Synergy H1), and viability for each condition was expressed as a percentage of the control group, which was defined as 100%.

### 2.5 Western blotting

Cells were harvested and lysed in RIPA buffer (50 mM Tris-HCl pH 7.4, 150 mM NaCl, 1% NP-40, 0.5% sodium deoxycholate, 0.1% SDS) supplemented with protease and phosphatase inhibitor cocktails (Roche, 04693159001 and 04906837001, respectively). Protein lysates were quantified using the BCA Protein Assay Kit (Thermo Scientific, 23225), and equal amounts of protein (30 μg per lane) were resolved by 8–12% SDS-PAGE and transferred onto PVDF membranes (Millipore, IPVH00010). Membranes were blocked for 1 h at room temperature with either 5% non-fat milk (for detection of non-phosphorylated targets) or 5% BSA (for phospho-specific antibodies) in TBST (Tris-buffered saline containing 0.1% Tween-20), followed by overnight incubation at 4 °C with primary antibodies. The following antibodies were used: anti-HIF-2α (NB100-122, Novus, 1:500), anti-phospho-HIF-2α (Ser672, PA5-104945, Thermo Fisher, 1:500), anti-phospho-AMPKα (Thr172, #2535, CST, 1:1000), anti-AMPKα (#5831, CST, 1:1000), anti-phospho-mTOR (Ser2448, #5536, CST, 1:1000), anti-mTOR (#2983, CST, 1:1000), anti-EPOR (ab123315, Abcam, 1:500), anti-γ-globin (sc-21756, Santa Cruz, 1:500), anti-Cleaved Caspase-3 (Asp175, #9664, CST, 1:500), anti-Caspase-3 (#9662, CST, 1:500), anti-Bax (#2772, CST, 1:500), anti-Bcl-2 (#15071, CST, 1:500), anti-DMT1 (ab55795, Abcam, 1:500), anti-FPN1 (ab235090, Abcam, 1:500), anti-FTH1 (ab75973, Abcam, 1:1000), and anti-β-actin (#4970, CST, 1:2000). After three washes with TBST, membranes were incubated with HRP-conjugated secondary antibodies (CST, 1:5000) for 1 h at room temperature. Immunoreactive bands were visualized using enhanced chemiluminescence (ECL; Millipore, WBKLS0500), and densitometric analysis was performed using ImageJ software (NIH, v1.53). Band intensities were normalized to β-actin or H3 as indicated, and results were expressed as fold change relative to the control group. For phospho-protein detection, blots were stripped in stripping buffer (62.5 mM Tris-HCl pH 6.8, 2% SDS, 100 mM β-mercaptoethanol) at 50 °C for 30 min and re-probed with total protein antibodies.

### 2.6 Cycloheximide chase assay for HIF-2α half-life

To evaluate HIF-2α protein turnover, cells were treated with cycloheximide (CHX; Sigma-Aldrich, C7698) at a final concentration of 50 μg/mL to inhibit *de novo* protein synthesis. Cells were collected at 0, 2, 4, and 6 h post-CHX administration, and whole-cell lysates were prepared for immunoblotting with anti-HIF-2α and anti-β-actin antibodies. HIF-2α band intensities were normalized to β-actin at each time point, and the amount remaining was expressed as a percentage of the 0-h control (set at 100%). The protein half-life was then calculated by fitting the decay curves to a one-phase exponential decay model using nonlinear regression in GraphPad Prism 9.0.

### 2.7 Co-immunoprecipitation

Co-immunoprecipitation (Co-IP) was performed to examine the interaction between HIF-2α and VHL. Cells (1 × 10^7^ per condition) were lysed in non-denaturing NP-40 lysis buffer (50 mM Tris-HCl pH 7.4, 150 mM NaCl, 1% NP-40, 1 mM EDTA, 10% glycerol) supplemented with protease and phosphatase inhibitors. Lysates were pre-cleared with 20 μL of Protein A/G magnetic beads (Thermo Scientific, 88802) for 30 min at 4 °C. Next, 500 μg of total protein was incubated overnight at 4 °C under rotation with either 4 μg of anti-HIF-2α antibody (NB100-122, Novus) or 4 μg of normal rabbit IgG (2729S, Cell Signaling) as a negative control. Following overnight incubation, 30 μL of Protein A/G magnetic beads was added and incubated for an additional 4 h at 4 °C. The beads were then washed four times with high-salt wash buffer (50 mM Tris-HCl pH 7.4, 300 mM NaCl, 0.5% NP-40, 1 mM EDTA). Immunoprecipitated proteins were eluted by boiling in 2× SDS sample buffer for 10 min and subsequently analyzed by Western blotting with anti-VHL (2738S, Cell Signaling, 1:1000) and anti-HIF-2α antibodies. Input samples (5% of total lysate) were included as loading controls.

### 2.8 RNA extraction and quantitative real-time PCR

Total RNA was extracted using TRIzol reagent (Invitrogen, 15596026) following the manufacturer’s instructions. RNA concentration and purity were evaluated with a NanoDrop 2000 spectrophotometer (Thermo Scientific). Reverse transcription was carried out with 1 μg of total RNA using the PrimeScript RT Reagent Kit (Takara, RR037A) in a final volume of 20 μL. Quantitative PCR (qPCR) was then performed using TB Green Premix Ex Taq II (Takara, RR820A) on a QuantStudio 5 Real-Time PCR System (Applied Biosystems). The thermal profile consisted of an initial denaturation at 95 °C for 30 s, followed by 40 cycles of 95 °C for 5 s and 60 °C for 30 s, with a melting curve analysis included at the end to confirm the specificity of each amplicon. Relative mRNA expression levels were calculated using the 2^-^ΔΔCt method, with β-actin (ACTB) serving as the endogenous reference. Primer sequences are provided in **Table 1**.

**Table 1:** Primer sequences for qPCR.

Gene	Forward (5’→3’)	Reverse (5’→3’)	Product (bp)
EPOR	CTGGCCTGTGTGTGTGAGTA	AGGGTTCACAGCCTTCTCCT	112
SLC11A2 (DMT1)	ACTTTGGGAAGGCAACATCC	GACTGAGGAGGTTCCGACAG	128
SLC40A1 (FPN1)	TCCTGCATGATCCTGCCTAC	CAGCATAGGAGCCACAGTCC	134
ACTB (β-actin)	CACCATTGGCAATGAGCGGTTC	AGGTCTTTGCGGATGTCCACGT	106

### 2.9 Enzyme-linked immunosorbent assay for hepcidin

Hepcidin concentrations in culture supernatants were determined using a commercial ELISA kit (Abcam, ab273283) following the manufacturer’s instructions. Supernatants collected at 24 h were centrifuged (300 × g, 5 min) and stored at −80 °C. Samples were diluted 1:2 with diluent, and 50-μL aliquots were incubated in pre-coated wells with biotinylated detection antibody for 1 h at RT. After washing, HRP–streptavidin was applied for 30 min, followed by TMB substrate development and stop solution addition at 15 min. Absorbance at 450 nm was measured, and hepcidin levels were calculated against a recombinant standard curve. All samples were run in duplicate.

### 2.10 Statistical analysis

All experiments were repeated at least three times with independent biological replicates (n = 3). Results are shown as mean ± SD. Statistical significance was evaluated by two-tailed Student’s t-test for two-group comparisons or by one-way ANOVA with Tukey’s *post hoc* test for multiple comparisons, with P < 0.05 considered significant. GraphPad Prism 9.0 was used for all statistical analyses. For immunoblotting, densitometric signals were normalized to the control group prior to statistical testing.

## 3 Results

### 3.1 Establishment and characterization of an anemic stress model in UT-7/EPO cells

Anemic stress was modeled by culturing UT-7/EPO cells under hypoxia (1% O_2_) with low EPO (0.2 U/mL). As shown in **Figure 1A**, this resulted in a significant decline in viability to 55.3% ± 6.2% of control at 24 h (P < 0.01). Time-course analysis revealed progressive decreases at 6 h (12.0%), 12 h (31.7%), and 24 h (52.4%) (all P < 0.01; **Figure 1B**).

**Figure 1:**
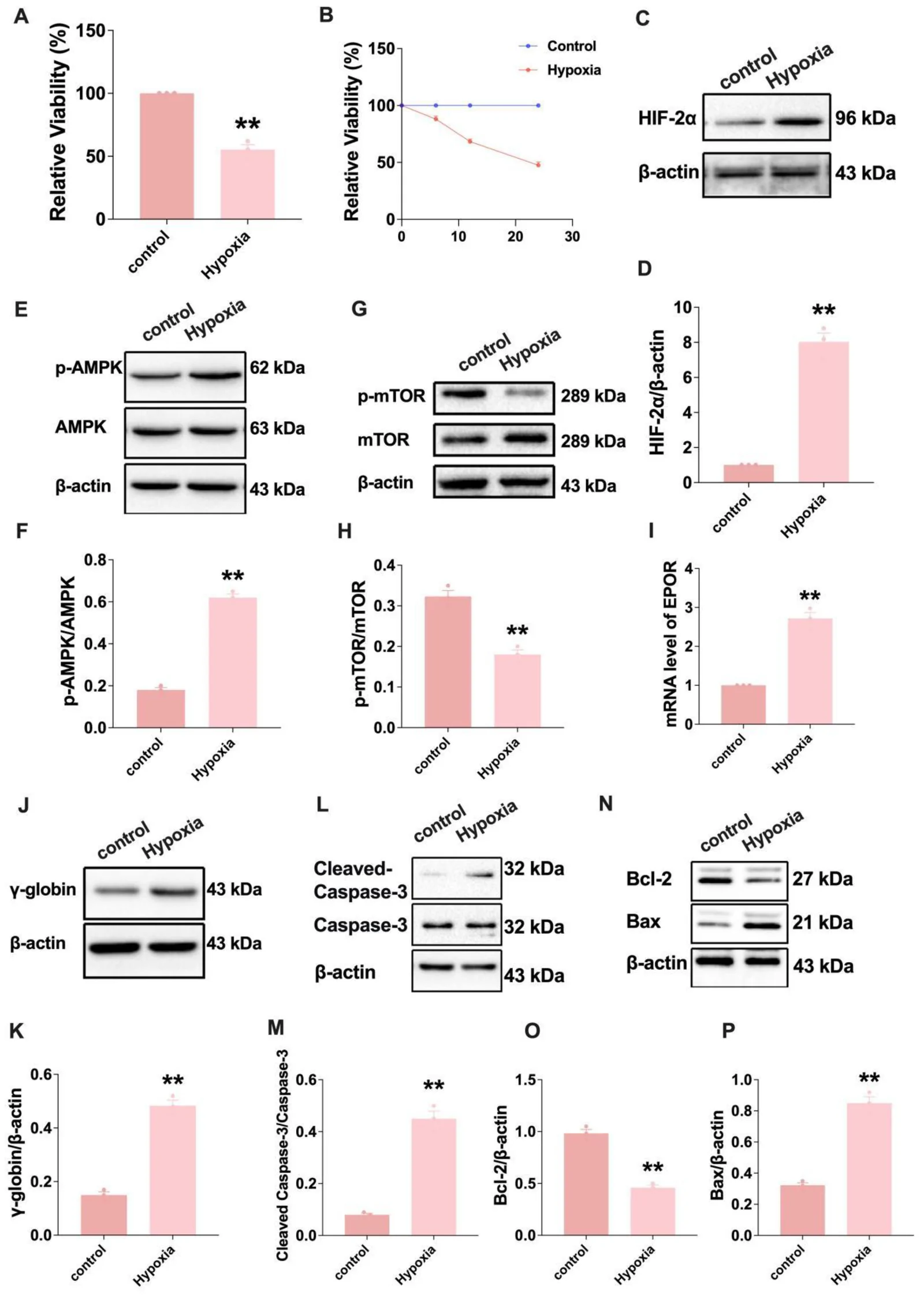
Establishment and characterization of an anemic stress model. UT-7/EPO cells were cultured under normoxia + normal EPO (control) or hypoxia + low EPO (hypoxia) for 24 h. **(A)** Cell viability (CCK-8). **(B)** Time-course of viability (0–24 h). **(C, D)** HIF-2α protein (Western blot). **(E–H)** p-AMPK and p-mTOR levels. **(I)** EPOR mRNA (qPCR). **(J, K)** γ-globin protein. **(L–P)** Cleaved Caspase-3 and Bax/Bcl-2 ratio. **P < 0.01 vs. control. Data are mean ± SD (n = 3). See Methods for detailed descriptions.

Western blot analysis demonstrated that anemic stress stabilized HIF-2α protein by approximately 8.0-fold (**Figures 1C, D**) and activated AMPK (p-AMPK/AMPK ratio increased 3.4-fold) while suppressing mTOR (p-mTOR/mTOR ratio decreased to 0.56-fold) (**Figures 1E–H**). Functionally, EPOR mRNA (**Figure 1I**) and γ-globin protein were upregulated by 2.7-fold and 3.2-fold, respectively (**Figures 1J, K**). Additionally, anemic stress induced apoptosis, as evidenced by a 5.6-fold increase in Cleaved Caspase-3 (**Figures 1L, M**) and a 5.6-fold increase in the Bax/Bcl-2 ratio (**Figures 1N–P**).

Collectively, these data confirm that the hypoxia + low EPO condition successfully recapitulates key features of anemic stress, including HIF-2α stabilization, AMPK activation with reciprocal mTOR suppression, induction of erythroid differentiation markers, and activation of apoptotic pathways.

### 3.2 AMPK activation stabilizes HIF-2α by disrupting VHL binding

To assess the impact of AMPK activation on HIF-2α stability, UT-7/EPO cells subjected to anemic stress were incubated with either the AMPK agonist AICAR or the inhibitor Compound C. CHX chase assay revealed that AICAR significantly prolonged HIF-2α half-life from 4.2 h (hypoxia alone) to 8.5 h, whereas Compound C reduced it to 2.1 h (**Figures 2A, B**). Consistently, AICAR increased HIF-2α protein levels by approximately 1.7-fold compared to hypoxia alone, while Compound C decreased them by approximately 47% (**Figures 2C, D**).

**Figure 2:**
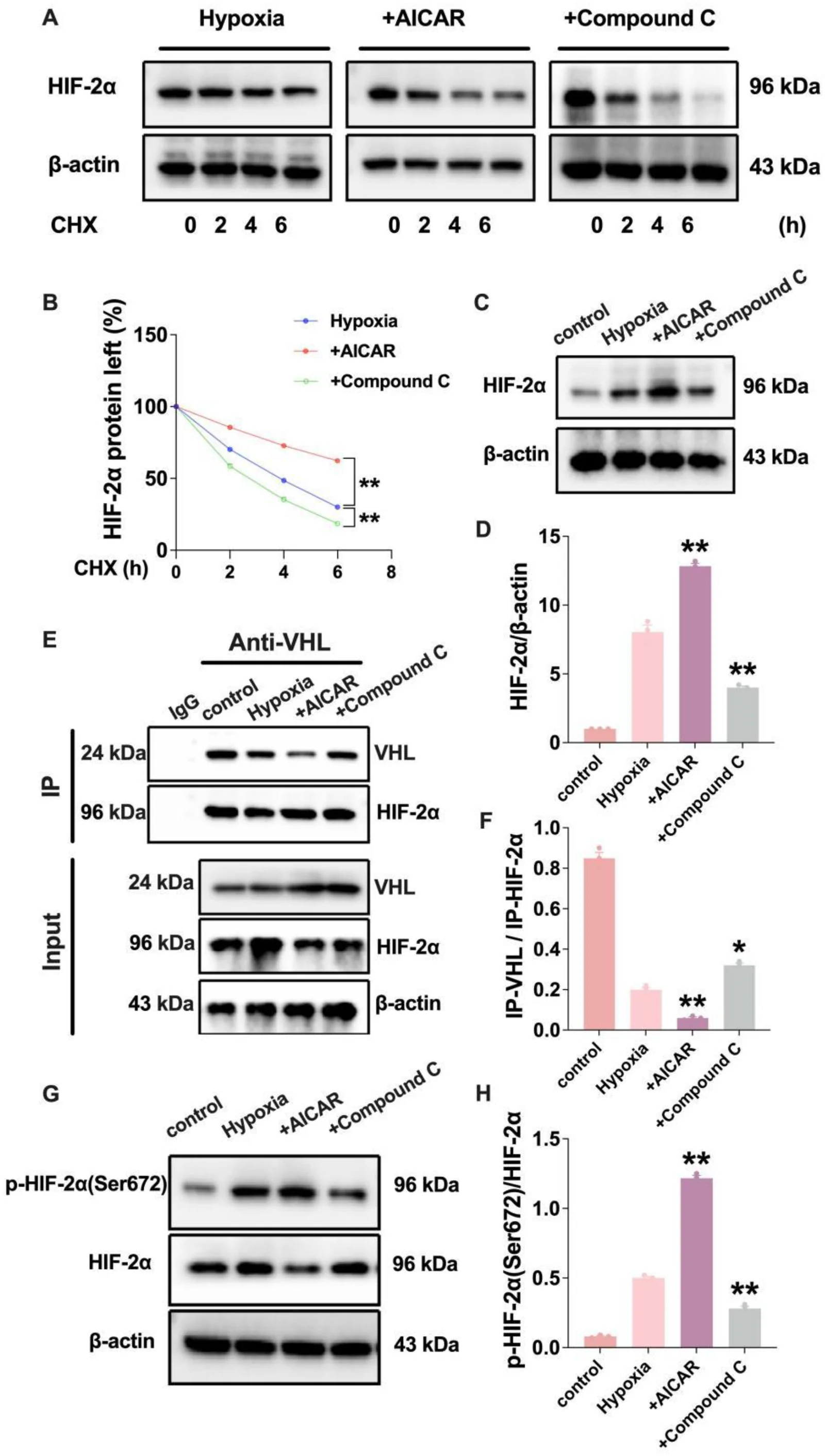
AMPK activation stabilizes HIF-2α by disrupting VHL binding. UT-7/EPO cells were treated under hypoxia + low EPO with or without AICAR (1 mM) or Compound C (10 μM) for 24 h. **(A, B)** CHX chase assay for HIF-2α half-life. Cells were harvested at 0, 2, 4, 6 h after CHX (50 μg/mL). Half-lives: Hypoxia 4.2 h, +AICAR 8.5 h, +CC 2.1 h. **(C, D)** HIF-2α protein expression (Western blot). β-actin as loading control. +AICAR ↑1.6-fold vs Hypoxia; +CC ↓50%. **(E, F)** Co-IP of HIF-2α-VHL interaction. IP: anti-HIF-2α; IB: anti-VHL. +AICAR disrupted VHL binding (ratio 1.00→0.30); +CC increased binding (1.60). **(G, H)** p-HIF-2α (Ser672) levels. +AICAR increased p-HIF-2α/HIF-2α ratio (0.50→1.22); +CC decreased it (0.28). *P < 0.05, **P < 0.01 vs. hypoxia group. Data are mean ± SD (n = 3).

Co-immunoprecipitation assays showed that AICAR markedly reduced the interaction between HIF-2α and VHL, with the VHL/input HIF-2α ratio decreasing from 0.21 (hypoxia) to 0.06 (P < 0.01). Conversely, Compound C partially restored VHL binding (ratio: 0.33) (**Figures 2E, F**). Notably, AICAR increased HIF-2α phosphorylation at Ser672, with the p-HIF-2α/HIF-2α ratio rising from 0.52 to 1.26 (P < 0.01), while Compound C reduced it to 0.28 (**Figures 2G, H**).

These results demonstrate that AMPK activation stabilizes HIF-2α by promoting Ser672 phosphorylation, which disrupts VHL binding and protects HIF-2α from proteasomal degradation.

### 3.3 The mTOR pathway mediates AMPK-induced HIF-2α stabilization

To determine whether mTOR acts downstream of AMPK to regulate HIF-2α stability, UT-7/EPO cells were treated with the mTOR inhibitor Rapamycin or activator MHY1485 under anemic stress. CHX chase assay revealed that Rapamycin prolonged HIF-2α half-life to 7.8 h, whereas MHY1485 reduced it to 1.5 h (**Figures 3A, B**). Consistently, Rapamycin increased HIF-2α protein levels by 1.6-fold compared to hypoxia alone, while MHY1485 decreased them by 55% (**Figures 3C, D**).

**Figure 3:**
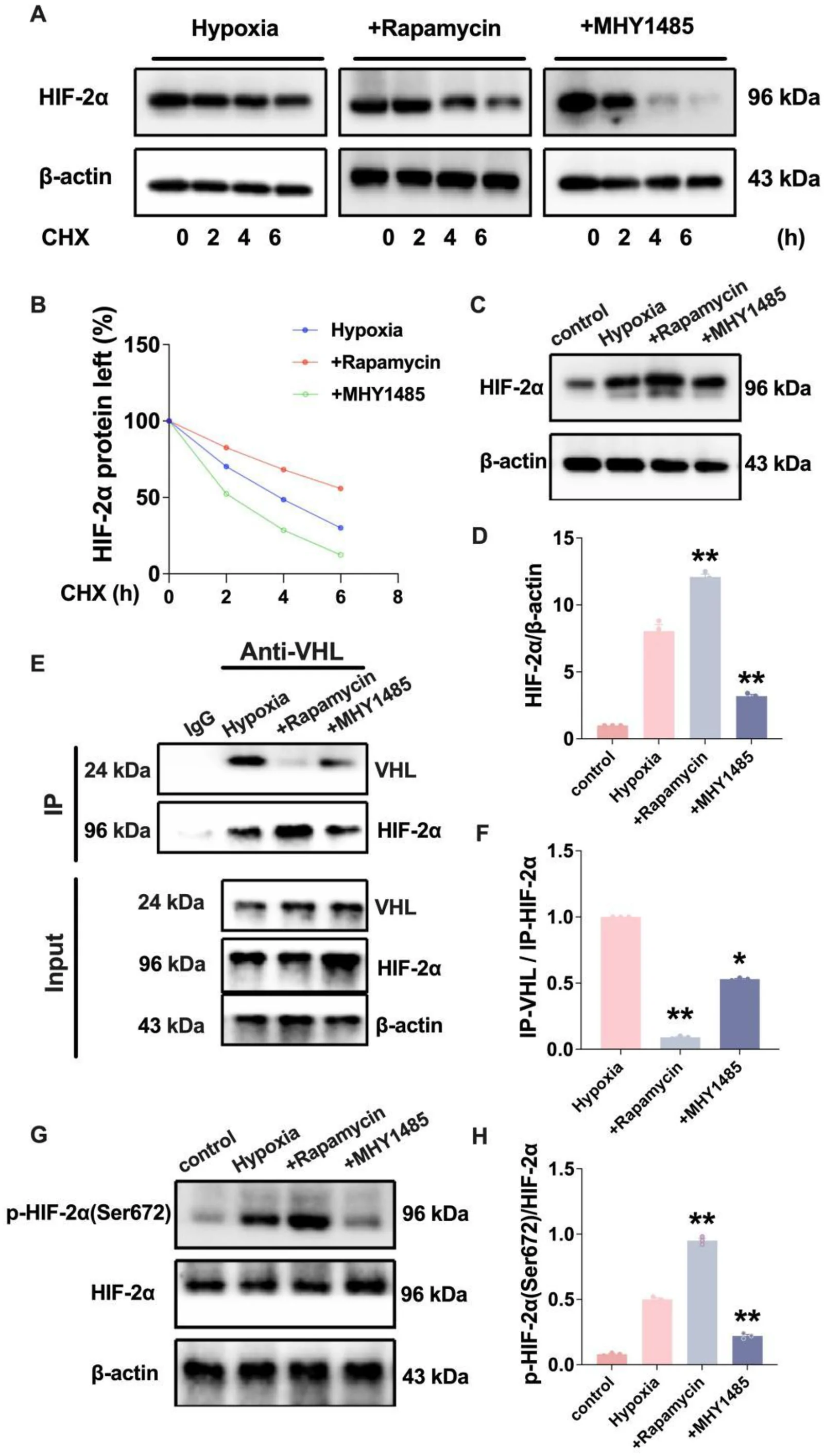
The mTOR pathway mediates AMPK-induced HIF-2α stabilization. UT-7/EPO cells were treated under hypoxia + low EPO with Rapamycin (100 nM, mTOR inhibitor) or MHY1485 (5 μM, mTOR activator) for 24 h. **(A, B)** CHX chase assay for HIF-2α half-life. Half-lives: Hypoxia 4.2 h, +Rapa 7.8 h, +MHY 1.5 h. **(C, D)** HIF-2α protein expression (Western blot). +Rapa ↑1.6-fold vs Hypoxia; +MHY ↓55%. **(E, F)** Co-IP of HIF-2α-VHL interaction. +Rapa disrupted VHL binding (ratio 1.00→0.09); +MHY increased binding (0.53). **(G, H)** p-HIF-2α (Ser672) levels. +Rapa increased p-HIF-2α/HIF-2α ratio (0.50→0.98); +MHY decreased it (0.22). *P < 0.05, **P < 0.01 vs. hypoxia group. Data are mean ± SD (n = 3).

Co-IP assays showed that Rapamycin markedly reduced HIF-2α-VHL interaction (normalized ratio: 1.00 → 0.09, P < 0.01), whereas MHY1485 increased it to 0.53 (**Figures 3E, F**). Notably, Rapamycin increased HIF-2α phosphorylation at Ser672 (p-HIF-2α/HIF-2α ratio: 0.50 → 0.98, P < 0.01), while MHY1485 reduced it to 0.22 (**Figures 3G, H**).

These results indicate that mTOR activation opposes AMPK-mediated HIF-2α stabilization, confirming that AMPK modulates HIF-2α stability through the mTOR pathway.

### 3.4 AMPK/mTOR pathway regulates HIF-2α target gene expression

To assess functional consequences of AMPK/mTOR-mediated HIF-2α stabilization, we examined HIF-2α target genes involved in erythropoiesis and iron metabolism.

qPCR analysis revealed that AICAR significantly upregulated EPOR, DMT1, and FPN1 mRNA by 6.2-, 4.5-, and 5.1-fold vs hypoxia (all P < 0.01), whereas Compound C suppressed them to near-baseline levels. Rapamycin similarly upregulated these genes (5.8-, 4.1-, and 4.8-fold), while MHY1485 suppressed them (0.9-, 0.6-, and 0.7-fold) (**Figures 4A–C**).

**Figure 4:**
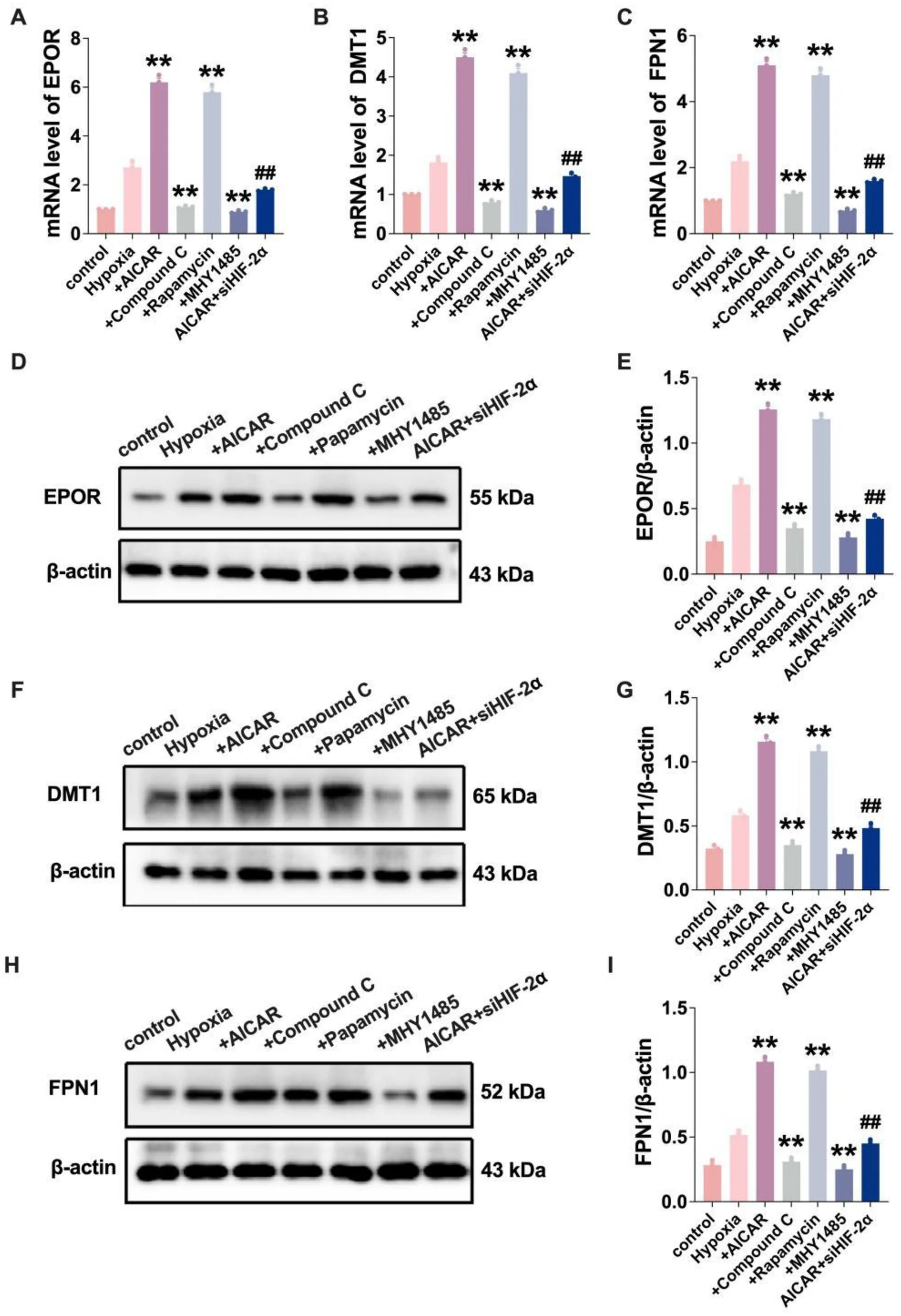
AMPK/mTOR pathway regulates HIF-2α target gene expression. UT-7/EPO cells were treated as indicated for 24 h. **(A–C)** qPCR analysis of EPOR **(A)**, DMT1 **(B)**, and FPN1 **(C)** mRNA. Data shown as fold change vs Control (mean ± SD, n = 3). **P < 0.01 vs hypoxia; ^##^P < 0.01 vs AICAR. **(D–I)** Representative Western blots **(D, E)** and densitometric analysis of EPOR **(F, G)** and DMT1/FPN1 **(H, I)**. β-actin as loading control. **P < 0.01 vs hypoxia; ^##^P < 0.01 vs AICAR. AICAR and Rapamycin upregulated target genes; MHY1485 and Compound C suppressed them. HIF-2α knockdown (siHIF-2α) abolished AICAR-induced upregulation.

AICAR treatment markedly increased EPOR (~5.0-fold), DMT1 (~4.2-fold), and FPN1 (~4.8-fold) protein expression relative to hypoxia (all P < 0.01), as determined by Western blotting. These effects were abrogated by either MHY1485 co-treatment or HIF-2α silencing (**Figures 4D–I**), indicating HIF-2α dependency.

These results demonstrate that AMPK activation enhances HIF-2α target gene expression via the mTOR pathway.

### 3.5 AMPK activation improves iron metabolism in a HIF-2α-dependent manner

Hepcidin secretion, assessed by ELISA, was significantly upregulated by hypoxia (25.3 ± 3.1 vs. 10.2 ± 1.5 ng/mL for control; P < 0.01). AICAR attenuated this response (12.4 ± 1.8 ng/mL; P < 0.01 vs. hypoxia), whereas Compound C increased it further (38.6 ± 4.2 ng/mL). Rapamycin also reduced hepcidin (15.3 ± 2.1 ng/mL), while MHY1485 increased it (45.2 ± 5.1 ng/mL) (**Figure 5A**).

**Figure 5:**
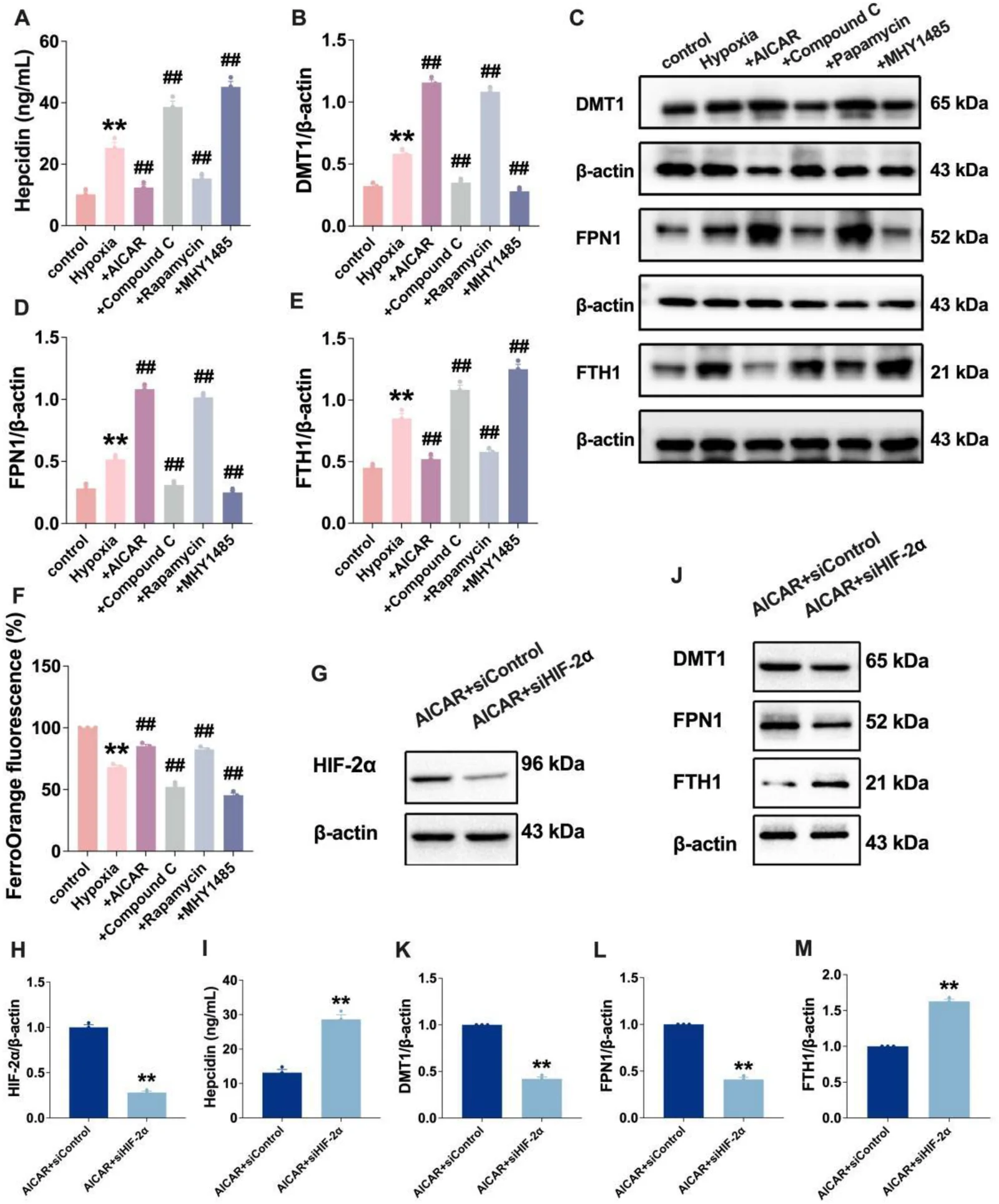
AMPK activation improves iron metabolism in a HIF-2α-dependent manner. UT-7/EPO cells were treated as indicated for 24 h. **(A)** Hepcidin concentration in culture supernatants (ELISA). Data shown as mean ± SD (n = 3). **P < 0.01 vs control; ^##^P < 0.01 vs hypoxia. **(B–D)** Representative Western blots and densitometric analysis of DMT1, FPN1 and Ferritin (FTH1). β-actin as loading control. **P < 0.01 vs control; ^##^P < 0.01 vs hypoxia. **(E)** FTH1 expression (Western blot). **P < 0.01 vs control; ^##^P < 0.01 vs hypoxia. **(F)** Intracellular labile iron pool (FerroOrange fluorescence). Data as % of control. **P < 0.01 vs control; ^##^P < 0.01 vs hypoxia. **(G–M)** Rescue experiment with HIF-2α knockdown. **(G, H)** Western blot confirming HIF-2α knockdown efficiency (>70% reduction). **(I)** Hepcidin levels (ELISA). **(J–M)** Densitometric analysis of DMT1, FPN1, and ferritin expression. **P < 0.01 vs AICAR + siControl.

Western blot analysis showed that AICAR upregulated DMT1 and FPN1 proteins by approximately 2.0-fold vs hypoxia (both P < 0.01), while Compound C and MHY1485 suppressed them (**Figures 5B–D**). Conversely, ferritin (iron storage) was elevated under hypoxia (0.85 ± 0.07 vs control 0.45 ± 0.04) and reduced by AICAR (0.52 ± 0.05, P < 0.01) (**Figure 5E**).

Intracellular labile iron levels (FerroOrange assay) decreased under hypoxia (68% of control) and were partially restored by AICAR (85%) but further reduced by Compound C (52%) (**Figure 5F**).

HIF-2α knockdown abolished AICAR-induced hepcidin suppression, DMT1/FPN1 upregulation, and ferritin reduction (**Figures 5G–M**), confirming that these effects are HIF-2α-dependent.

### 3.6 Integrated model of the AMPK/mTOR/HIF-2α axis in stress erythropoiesis

Based on the findings from **Figures 1**–**5**, we propose a working model for the AMPK/mTOR/HIF-2α axis in stress erythropoiesis (**Figure 6A**). Under anemic stress (hypoxia + low EPO), AMPK is activated, leading to increased phosphorylation of HIF-2α at Ser672. This phosphorylation disrupts HIF-2α-VHL binding, reduces HIF-2α ubiquitination, and stabilizes the protein. Stabilized HIF-2α upregulates EPOR (enhancing EPO sensitivity), DMT1 and FPN1 (improving iron import/export), while suppressing hepcidin (releasing iron from stores). The mTOR pathway acts downstream of AMPK, with mTOR activation opposing AMPK-mediated HIF-2α stabilization (**Figure 6B**).

**Figure 6:**
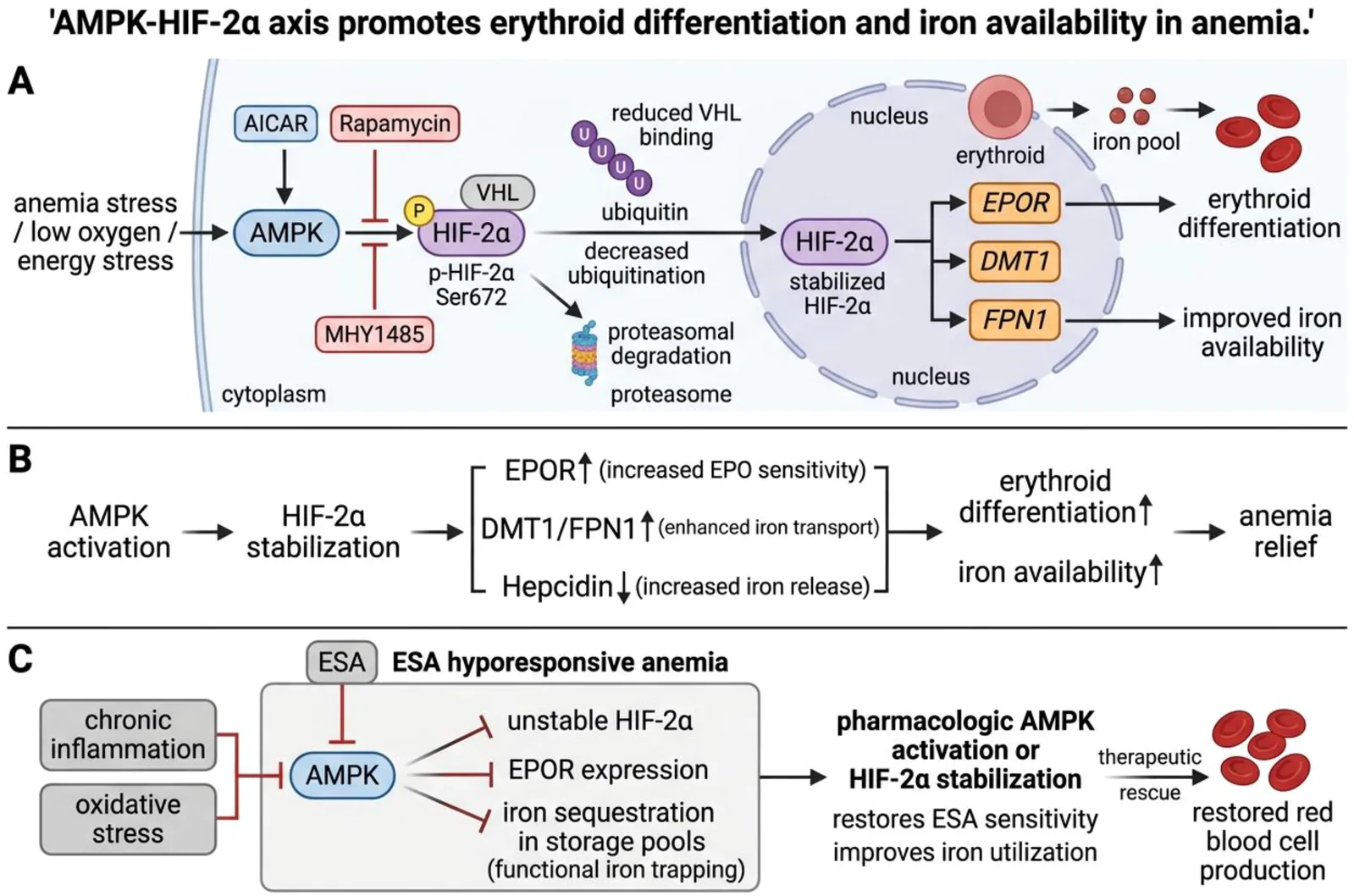
Integrated model and clinical implications of the AMPK/mTOR/HIF-2α axis. **(A)** Molecular mechanism model. Anemic stress activates AMPK, promoting HIF-2α Ser672 phosphorylation, which disrupts VHL binding, reduces ubiquitination, and stabilizes HIF-2α. Stabilized HIF-2α upregulates EPOR, DMT1, and FPN1 while suppressing hepcidin, enhancing erythropoiesis and iron availability. mTOR activation opposes AMPK-mediated HIF-2α stabilization. **(B)** Functional overview diagram. AMPK activation → HIF-2α stabilization → EPOR↑ (EPO sensitivity), DMT1/FPN1↑ (iron transport), hepcidin↓ (iron release) → erythroid differentiation↑ and iron availability↑. **(C)** Clinical implications. In ESA-hyporesponsive anemia, inflammation/oxidative stress may impair AMPK signaling. Pharmacological AMPK activation (metformin/AICAR) or HIF-2α stabilization may restore ESA sensitivity and improve iron utilization.

These findings have potential clinical implications for ESA-hyporesponsive anemia. In patients with chronic inflammation or oxidative stress, impaired AMPK signaling may lead to reduced HIF-2α stability, decreased EPOR expression, and iron sequestration (elevated hepcidin and ferritin). Pharmacological activation of AMPK (e.g., with metformin or AICAR) or direct HIF-2α stabilization may represent novel therapeutic strategies to restore ESA sensitivity and improve iron utilization (**Figure 6C**).

A summary of all key findings across the six experimental groups is presented in **Table 2** (see table below).

**Table 2:** Summary table of key findings across all experimental groups.

	Control	Hypoxia	+AICAR	+CC	+Rapa	+MHY
HIF-2α protein	—	REF	↑↑↑	↑	↑↑↑	→
p-AMPK/AMPK	—	REF	↑↑↑	↓	↑	→
p-mTOR/mTOR	—	REF	↓↓	↑	↓↓	↑↑
p-HIF-2α/HIF-2α	—	REF	↑↑	↓	↑	↓
EPOR mRNA/protein	—	REF	↑↑↑	→	↑↑	↓
DMT1 mRNA/protein	—	REF	↑↑	↓	↑↑	↓
FPN1 mRNA/protein	—	REF	↑↑	↓	↑↑	↓
Hepcidin	—	REF	↓	↑↑↑	↓	↑↑↑
Ferritin	—	REF	↓	↑↑	↓	↑↑↑
Intracellular Iron	—	REF	↑	↓↓	↑	↓↓
γ-globin	—	REF	↑↑	→	↑↑	↓

Data are expressed as relative changes compared to the Hypoxia group (set as reference). ↑, mild increase (1.5–3.0-fold); ↑↑, moderate increase (3.0–6.0-fold); ↑↑↑, marked increase (>6.0-fold); ↓, mild decrease (0.5–0.7-fold); ↓↓, marked decrease (<0.5-fold); →, no significant change (0.8–1.2-fold); —, no significant change; n.d., not determined. See Results for detailed quantitative data.

## 4 Discussion

This study is the first to reveal that AMPK activation stabilizes HIF-2α by attenuating its interaction with VHL, thereby facilitating erythroid maturation and counteracting dysregulated iron metabolism during anemic stress, with these effects occurring via the mTOR signaling cascade. Using UT-7/EPO cells as a model of anemic stress, we demonstrate that pharmacological activation of AMPK with AICAR prolongs HIF-2α half-life from 4.2 h to 8.5 h, increases HIF-2α Ser672 phosphorylation, disrupts the HIF-2α-VHL interaction, and reduces HIF-2α ubiquitination, leading to enhanced expression of EPOR and iron-regulatory genes DMT1 and FPN1, as well as suppression of hepcidin. Conversely, AMPK inhibition or mTOR activation promotes HIF-2α degradation and attenuates these pro-erythroid effects. Together, our results position the AMPK/mTOR/HIF-2α regulatory network as a key mediator of stress-induced erythroid regeneration, and raise the prospect that targeting this pathway could offer a viable alternative for anemia management, with particular relevance to ESA-refractory patient populations.

A key mechanistic insight from our study is that AMPK activation promotes HIF-2α phosphorylation at Ser672, which correlates with reduced VHL binding and increased protein stability. This finding extends previous work demonstrating that AMPK directly phosphorylates HIF-1α at Ser-777/Ser-778 under hypoxic conditions, enhancing its transcriptional activity independently of oxygen tension (Moulin et al., 2025). HIF-2α Ser672 is homologous to HIF-1α Ser652, and both are located within the oxygen-dependent degradation domain (ODDD) that governs VHL binding (Maxwell et al., 1999; Kaelin et al., 2008). Phosphorylation at this site may induce conformational changes that mask the VHL recognition motif, thereby preventing ubiquitination and proteasomal degradation. Although we did not perform *in vitro* kinase assays to directly confirm AMPK-mediated HIF-2α phosphorylation, several lines of evidence support this interpretation: (1) AICAR increased HIF-2α Ser672 phosphorylation, while Compound C decreased it; (2) the AICAR-induced alterations in Ser672 phosphorylation and VHL association were reversed by MHY1485 co-treatment; and (3) HIF-2α knockdown abolished the functional effects of AICAR on target gene expression and iron metabolism.

These findings further suggest that AMPK modulates HIF-2α stability, at least in part, via the mTOR pathway. Rapamycin, an mTOR inhibitor, mimicked the effects of AMPK activation by prolonging HIF-2α half-life to 7.8 h, reducing VHL binding, and increasing Ser672 phosphorylation. Conversely, MHY1485, an mTOR activator, opposed AMPK-mediated HIF-2α stabilization and partially reversed the effects of AICAR. These findings corroborate the known role of AMPK as a suppressor of mTORC1 (Inoki et al., 2012). However, the exact mechanism by which mTOR signaling regulates HIF-2α stability is not yet fully understood. One possibility is that mTORC1 regulates the translation or activity of PHD enzymes, which control HIF-α hydroxylation and subsequent VHL binding (Xue et al., 2023). Alternatively, mTORC2, which is insensitive to rapamycin, may modulate HIF-2α through AKT-dependent signaling (Sarbassov et al., 2005). Given that MHY1485 activates both mTORC1 and mTORC2 (Yu et al., 2025), distinguishing the contributions of these two complexes will require further investigation using isoform-selective inhibitors or genetic approaches.

From a functional perspective, AMPK-mediated HIF-2α stabilization resulted in enhanced expression of EPOR, FPN11, and DMT1, as well as reduced hepcidin and ferritin levels. These effects are collectively pro-erythroid. Increased EPOR expression may sensitize erythroid progenitors to residual EPO, which is particularly relevant in anemic patients with elevated EPO levels but poor response (Cooper et al., 2003; Cen et al., 2022). Improved iron import via DMT1 and export via FPN1 facilitates hemoglobin synthesis, while reduced hepcidin prevents iron sequestration in macrophages and hepatocytes (Mastrogiannaki et al., 2009; Kapitsinou et al., 2010; Ganz, 2011). Of note, our observation that AICAR decreased hepcidin secretion (from 25.3 to 12.4 ng/mL) in UT-7/EPO cells corroborates previous findings that HIF-2α suppresses hepcidin gene expression by competing with C/EBPα for promoter occupancy (Mastrogiannaki et al., 2013). The observation that AICAR also reduced ferritin levels (from 0.85 to 0.52) and partially restored intracellular labile iron levels (from 68% to 85% of control) provides direct evidence that AMPK activation improves iron mobilization from storage pools.

IInterestingly, the anemic stress condition also activated pro-apoptotic pathways, as indicated by upregulation of Cleaved Caspase-3 and a higher Bax/Bcl-2 ratio. Although AICAR treatment partially reduced apoptosis (data not shown), the persistent elevation of apoptotic markers suggests that while AMPK activation promotes erythroid differentiation, it does not completely rescue cell death under severe anemic stress. This observation is consistent with the concept of “stress erythropoiesis,” where accelerated differentiation occurs at the expense of progenitor expansion (Paulson et al., 2011). The balance between survival, proliferation, and differentiation under anemic stress likely involves additional signaling pathways beyond the AMPK/mTOR/HIF-2α axis, such as JAK-STAT and NF-κB (Richmond et al., 2005; Wong and Sugimura, 2023).

Our findings have potential clinical implications for patients with ESA hyporesponsiveness, a condition affecting up to 10% of anemic patients, particularly those with chronic kidney disease, heart failure, or inflammatory disorders (Macdougall and Cooper, 2002; Okonko et al., 2013). These patients typically exhibit elevated inflammatory cytokines (TNF-α, IL-6) and oxidative stress, which may impair AMPK signaling (Stenvinkel and Bárány, 2002; Khalil et al., 2016). We speculate that in such patients, reduced AMPK activity leads to decreased HIF-2α stability, lower EPOR expression, and iron sequestration (elevated hepcidin and ferritin), contributing to ESA resistance. Pharmacological activation of AMPK with agents such as metformin (a widely used anti-diabetic drug with known AMPK-activating properties) has been associated with reduced ESA requirements in diabetic CKD patients (Inzucchi et al., 2015), raising the intriguing possibility that AMPK activation could improve ESA responsiveness. However, it should be noted that metformin has pleiotropic effects beyond AMPK activation, and its role in anemia management requires prospective clinical validation.

Our findings also complement the mechanism of action of HIF-PHD inhibitors (e.g., roxadustat), which are approved for treatment of renal anemia in several countries (Ha et al., 2024). While HIF-PHD inhibitors stabilize HIF-α by blocking its hydroxylation, our study identifies AMPK as an upstream regulator that stabilizes HIF-2α through a distinct mechanism—phosphorylation-dependent disruption of VHL binding. This raises the possibility that combining AMPK activators with HIF-PHD inhibitors may produce synergistic effects, potentially allowing lower doses of each agent and reducing side effects such as polycythemia or pulmonary hypertension (Ha et al., 2024). Nevertheless, caution is warranted, as excessive HIF-2α stabilization has been linked to adverse outcomes. The therapeutic window for AMPK-targeted interventions in anemia may be narrow, and future studies should explore intermittent or cell-type-specific targeting strategies.

## 5 Conclusions

In summary, this study establishes the AMPK/mTOR/HIF-2α axis as a critical regulator of stress erythropoiesis. We demonstrate that AMPK activation stabilizes HIF-2α by promoting Ser672 phosphorylation, which disrupts VHL binding, reduces ubiquitination, and protects HIF-2α from proteasomal degradation. This leads to enhanced expression of EPOR, FPN1, and DMT1, as well as suppression of hepcidin and ferritin, collectively promoting erythroid differentiation and improving iron availability under anemic stress. These findings provide a mechanistic basis for developing AMPK-targeted therapies for anemia, particularly in ESA-hyporesponsive patients, and suggest that the AMPK/mTOR/HIF-2α axis constitutes a promising therapeutic target for disorders affecting erythropoiesis and iron homeostasis.

## Data Availability

The original contributions presented in the study are included in the article/supplementary material, further inquiries can be directed to the corresponding author/s.
